# Factors influencing the outcomes of minimally invasive total hip arthroplasty: a systematic review

**DOI:** 10.1186/s13018-022-03168-4

**Published:** 2022-05-18

**Authors:** Filippo Migliorini, Andrea Pintore, Joerg Eschweiler, Francesco Oliva, Frank Hildebrand, Nicola Maffulli

**Affiliations:** 1 Department of Orthopaedic, Trauma, and Reconstructive Surgery, RWTH University Hospital, Pauwelsstraße 30, 52074 Aachen, Germany; 2 Department of Medicine, Surgery and Dentistry, University of Salerno, Via S. Allende, 84081 Baronissi, SA Italy; 3 School of Pharmacy and Bioengineering, Keele University School of Medicine, Thornburrow Drive, Stoke on Trent, England UK; 4 Barts and the London School of Medicine and Dentistry, Centre for Sports and Exercise Medicine, Queen Mary University of London, Mile End Hospital, 275 Bancroft Road, London, E1 4DG England UK

**Keywords:** Hip, Arthroplasty, Replacement, Minimally invasive

## Abstract

**Introduction:**

The present systematic review investigated possible factors which may influence the surgical outcome of minimally invasive surgery for total hip arthroplasty (MIS THA).

**Methods:**

In January 2022, the Embase, Google Scholar, PubMed, and Scopus databases were accessed. All the clinical trials investigating the clinical outcome of MIS THA were considered.

**Results:**

Data from 9486 procedures were collected. Older age was moderately associated with greater Visual Analogue Scale (VAS) (*P* = 0.02) and Western Ontario and McMaster Universities Osteoarthritis Index (WOMAC) (*P* = 0.009) at last follow-up, and shorter surgical duration (*P* = 0.01). Greater body mass index (BMI) at baseline was moderately associated with greater cup anteversion (*P* = 0.0009), Oxford Hip Score (OHS) at last follow-up (*P* = 0.04), longer surgical duration (*P* = 0.04), increased leg length discrepancy (*P* = 0.02), and greater rate of infection (*P* = 0.04). Greater VAS at baseline was weakly associated with greater VAS at last follow-up (*P* < 0.0001), total estimated blood lost (*P* = 0.01), and lower value of Harris Hip Score (HHS) (*P* = 0.0005). Greater OHS at baseline was associated with greater post-operative VAS (*P* = 0.01). Greater WOMAC at baseline was associated with lower cup anteversion (*P* = 0.009) and greater VAS (*P* = 0.02). Greater HHS at baseline was associated with shorter hospitalisation (*P* = 0.001).

**Conclusion:**

Older age and greater BMI may represent negative prognostic factors for MIS THA. The clinical outcome is strongly influenced by the preoperative status of patients.

## Introduction

Minimally invasive surgery (MIS) for total hip arthroplasty (THA) has become popular [1]. The definition of MIS in THA is controversial. Currently, MIS surgery refers to a tissue sparring approach [2]; [3]. During the last decade, a variety of MIS approaches have been described. MIS THA has been introduced in an effort to speed recovery and decrease the length of hospitalisation [4–11]. MIS THA has been advocated to reduce the length of the surgical procedure, quadriceps damage, and the total estimated blood loss [12–16]. MIS THA can be performed in a single incision using the posterior [15]; [17], lateral [18]; [19], anterolateral [20] and anterior approach [21]. Also, multiple incisions MIS THA procedures have been described, such as the two-incision approach [22]; [23], and the minimally invasive anterior approach with accessory incision [21]. However, based on current available evidence, there are no clinically relevant benefits of MIS THA over traditional approaches in terms of functional outcome and components orientation, and MIS THA carries high rate of complications [12]; [15]; [24]; [25]. Nevertheless, MIS THA remains of special interest of patients and surgeons. To date, though the current literature includes several thousands of scientific reports, there is paucity of evidence concerning the role of prognostic factors for MIS THA. The goal of the present study was to investigate potential associations between the patient characteristics at admission, peri-operative data, imaging findings, and the clinical and functional outcome, and complications. A multiple linear regression analysis was conducted to identify possible prognostic factors which may influence the clinical outcome.

## Material and methods

### Search strategy

This systematic review followed the Preferred Reporting Items for Systematic Reviews and Meta-Analyses (PRISMA) [26]. The PICO algorithm was preliminarily set out:

- P (Population): end-stage OA;
- I (Intervention): MIS THA;
- C (Comparison): patients characteristics at admission;
- O (Outcomes): PROMs, radiological findings, complications;

### Data source and extraction

Two authors (F.M. and A.P.) independently performed the literature search in January 2022 accessing the following databases: PubMed, Google Scholar, Embase, and Scopus. The following keywords were used and combined for the search: *hip, total, arthroplasty, replacement, prosthesis, instrumentation, surgery, intervention, BMI, age, sex.* The resulting abstracts were screened by the two authors and, if of interest, the full-text was accessed. The bibliographies were also screened by hand. Disagreement was debated and solved by the senior author (N.M.).

### Eligibility criteria

All the clinical trials investigating the outcomes of MIS THA were accessed. Only studies comparing traditional versus MIS THA approaches were considered eligible. Given the authors languages capabilities, articles in English, German, Italian, French and Spanish were eligible. Only levels I to III of evidence, according to the Oxford Centre of Evidence-Based Medicine [27], were eligible. Reviews, letters, opinions, editorials, and technical notes were not considered, nor were abstracts and national registries. Animal, computational, biomechanics, cadaveric studies were not eligible. Studies reporting results from experimental surgeries and/ or pre- and/ or post-operative protocols were not included. Only articles reporting quantitative data under the outcomes of interest were considered for inclusion. Missing data under the outcomes of interest warranted the exclusion from this study.

### Outcomes of interest

Two authors (F.M. and A.P.) independently performed data extraction. Study generalities (author, year, journal, study design, length of the follow-up) were collected. Data concerning the following endpoints at baseline were collected:

- Patient demographics: number of procedures, mean BMI and age, percentage of female;
- PROMs: Visual Analogue Scale (VAS), Oxford Hip Score (OHS), The Western Ontario and McMaster Universities Osteoarthritis Index (WOMAC), Harris Hip Score (HHS).
  The present study investigated whether the aforementioned endpoints were associated with the outcome. Thus, every single endpoint was independently analysed, and its association with the following data at last follow-up assessed:
- Peri-operative data: surgical duration, total estimated blood loss, and length of hospital stay;
- Radiographic measures: mean cup inclination and anteversion, mean stem alignment, and limb length discrepancy;
- PROMs: Visual Analogue Scale (VAS), Oxford Hip Score (OHS), The Western Ontario and McMaster Universities Osteoarthritis Index (WOMAC), Harris Hip Score (HHS);
- Complications: dislocations, revisions, deep infections, aseptic loosening, fractures.

### Methodology quality assessment

The methodological quality assessment was made by two independent reviewers (F.M. and A.P.). The risk of bias graph tool of the Review Manager Software 5.3 (The Nordic Cochrane Collaboration, Copenhagen) was used. The following risk of bias was assessed for each included study: selection, detection, attrition, reporting, and other source of bias.

### Statistical analysis

The statistical analyses were performed by the main author (F.M.). For the analytical statistics, STATA MP 16 software (StataCorp, College Station, TX) was used. The Shapiro–Wilk test was performed to investigate data distribution. For normal data, mean and standard deviation (SD) were calculated. For nonparametric data, median and interquartile range (IQR) were calculated. A multivariate analysis was performed through a multiple pairwise correlations according to the Pearson product-moment correlation coefficient $\left( r \right)$. According to the Cauchy–Schwarz inequality, the final effect ranks between + 1 (positive linear correlation) and − 1 (negative linear correlation). Values of 0.1 < $\left| { r} \right|$< 0.3 and 0.3 < $\left| { r} \right|$< 0.5 and $\left| { r} \right|$> 0.5 were considered to have poor, moderate and strong correlation, respectively. Potential associations between one the endpoints and the outcomes of interest were evaluated singularly for each endpoint. Overall significance was evaluated using the *χ*^2^ test, with values of *P* > 0.05 considered statistically significant.

## Results

### Search result

**Fig. 1:**
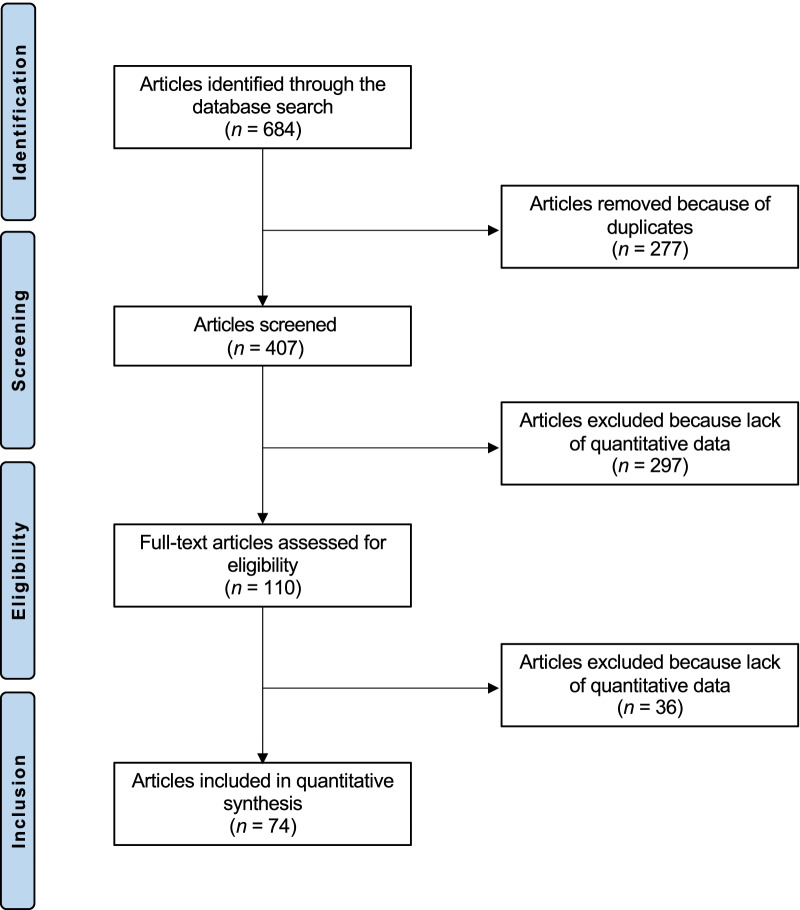
Flow chart of the literature search

The literature search resulted in 684 articles. Of them, 277 were excluded because they were duplicates. A further 297 articles were excluded since they did not match our eligibility criteria. Another 36 articles were not included because they did not report quantitative data under the outcomes of interest. This left 74 studies for the present study: 33 randomised, 29 prospective, and 17 retrospective studies. The literature search results are shown in Fig. 1.

### Methodological quality assessment

**Fig. 2:**
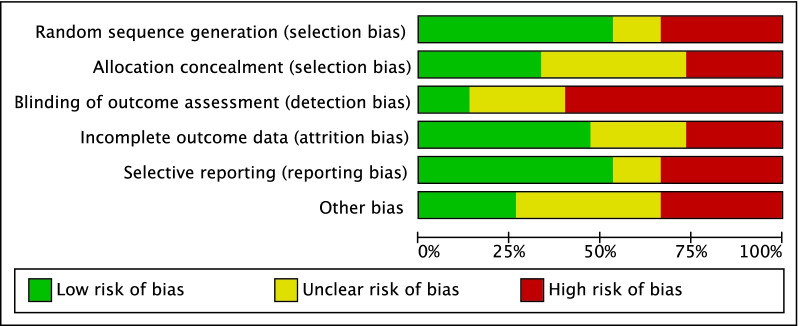
Methodological quality assessment

The risk of bias summary evidenced some limitations of the present study. Approximately half of the studies were randomised, and approximately one fifth were retrospective. This leads to a moderate risk of selection bias. Given the overall lack of blinding, the risk of detection bias was moderate-high. The authors' judgements about the risk of attrition, reporting and other bias presented across all included studies was moderate. Concluding, the overall risk of bias was moderate, attesting to this study good quality assessment (Fig. 2).

### Patient demographics

Data from 9626 procedures were collected. 57% (5487 of 9626 patients) were women. The median follow-up was 12 (IQR 9) months. The mean age was 63.0 (SD 4.9), the mean BMI 27.1 (SD 2.3) kg/m^2^. Generalities and patient baseline of the included studies are shown in Table 1.

**Table 1:** Generalities and patients baseline of the included studies

Author	Journal	Design	Follow up (*months*)	Procedures (*n*)	Women (*%*)	Mean age	Mean BMI	Approach
Abdel et al. [7]	*Arthroplasty*	Randomised	102.0	36	44.4	66.0	30.0	P MIS
				35	45.7	66.0	30.0	2 incision
Alecci et al. [82]	*J Orthop Traumatol*	Retrospective	0.5	221	54.8	70.7		A MIS
				198	62.1	70.1		L
Barrett et al. [83]	*Arthroplasty*	Randomised	12.0	43	32.6	61.4	30.7	A
				44	56.8	63.2	29.1	P
Bennett et al. [11]	*Arthroplasty*	Prospective	12.0	43	58.1	66.1	29.6	Minimal invasive
				52	46.2	64.6	29.2	Total incision
Berend et al. [84]	*Bone Joint Surg*	Randomised	1.5	258	56.4	63.0	28.9	A (ASI)
				372	56.4	63.0	30.4	L MIS (LIDL)
Bergin et al. [60]	*Bone Joint Surg*	Prospective	1.0	29	66.0	68.8	26.3	A
				28	50.0	65.1	27.8	P
Berstock et al. [85]	*J Othopaedics*	Retrospective	37.0	116	56.0	71.4		L OMEGA
				152	71.1	74.5		P
Biau et al. [86]	*Int Orthop*	Randomised	0.3	105	56.2	68.0	25.0	P MIS
				102	59.8	66.0	25.0	P
Chen et al. [87]	*Arthroplasty*	Retrospective	24.0	83	44.6	53.5	24.5	MIS-2 THA
				83	50.6	55.0	25.3	Conventional THA
Cheng et al. [88]	*Arthroplasty*	Randomised	3.0	35	57.0	59.0	27.7	A
				37	53.0	62.5	28.3	P
Chimento et al. [13]	*Arthroplasty*	Randomised	24.0	28	42.9	67.2	25.2	8 cm incision
				32	59.4	65.6	24.8	15 cm incision
Della Valle et al. [89]	*Clin Orthop Rel Res*	Randomised	12.0	35	68.6	63.8	27.3	P MIS
				37	67.6	61.2	27.6	2 incision
Dienstknecht et al. [14]	*J Orthop Surg*	Randomised	3.0	55	60.0	61.9	27.6	A MIS
				88	53.4	61.3	30.1	L
DiGioia et al. [90]	*Arthroplasty*	Prospective	12.0	33	57.6	65.0	27.0	Mini-incision
				33	57.6	65.0	28.0	L
Dorr et al. [15]	*Bone Joint Surg*	Randomised	6.0	30	43.3	70.3	27.6	Mini-incision
				30	53.3	63.9	30.2	Long-incision
Downing et al. [91]	*Acta Orthop Scand*	Prospective	12.0	49	51.0	67.0		P
				51	58.8	65.0		L
Engdal et al. [92]	*Am J Phys Med Rehab*	Prospective	0.2	21	61.9	56.8	25.8	L
				19	42.1	55.5	26.7	P
				20	75.0	56.4	25.8	A
Fink et al. [93]	*Orthopäde*	Prospective	1.5	50	54.0	71.5	28.0	PL
				50	50.0	71.9	27.0	Mini-posterior
Fransen et al. [94]	*Acta Orthop Belg*	Retrospective	12.0	38	62.9	62.6	27.6	PL
				45	66.7	64.2	25.0	A
Goebel et al. [55]	*Int Orthop*	Retrospective	3.0	100	53.0	64.5	26.7	Minimal A
				100	58.0	67.0	28.6	L
Goosen et al. [95]	*Clin Orthop Rel Res*	Randomised	12.0	30	50.0	60.0	26.7	AL MIS
				30	56.7	62.0	26.8	PL
				30	50.0	60.0	26.4	PL MIS
				30	46.7	62.0	26.1	AL
Hananouchi et al. [96]	*Int J Med Robotics Comput Assist Surg*	Prospective	12.0	20	90.0	55.1	22.2	A MIS
				20	90.0	57.0	21.0	P MIS
Howell et al. [96]	*Orthop Clin N Am*	Prospective	0.5	50	32.0	59.8	26.2	MIS-AL
				57	52.6	62.3	28.8	AL
Ilchmann et al. [97]	*Orthop Rev*	Prospective	24.0	142	47.0	70.0	27.4	L
				113	47.0	70.0	27.4	A MIS
Ji et al. [98]	*Arthroplasty*	Prospective	37.9	99	45.5	51.0	24.3	P
				97	40.2	52.0	24.3	L
Joseph et al. [99]	*Arthroplasty Today*	Prospective	6.0	98	54.1	61.1	30.4	A
				69	50.7	62.9	30.7	P
Khan et al. [100]	*Bone Joint Surg*	Randomised	24.0	52	63.5	72.8	28.9	P
				48	50.0	72.3	28.5	Piriformis-sparing
Ki et al. [101]	*Clin Orthop Surg*	Retrospective	51.5	34	38.2	61.0	22.3	PL MIS
				26	26.9	57.5	21.6	2 incision
Kim et al. [102]	*Arthroplasty*	Randomised	26.4	35	24.3	55.6	25.6	PL MIS
				35	24.3	55.6	25.6	PL
Kiyama et al. [103]	*Arthroplasty*	Randomised	6.0	10	90.0	60.3	23.4	PL MIS
				10	80.0	63.8	23.5	PL
Krych et al. [104]	*Clin Orthop Rel Res*	Randomised	1.5	10	38.1	63.0	30.0	P MIS
				11	38.1	63.0	30.0	2 incision
Laffosse et al. [105]	*Rev Chir Orthop*	Prospective	6.0	58	39.7	55.0	25.0	AL MIS
				58	43.1	59.7	26.2	P
Lafosse et al. [106]	*Arch Orthop Trauma Surg*	Prospective	6.0	33	39.4	56.8	25.9	AL MIS
				43	34.9	55.7	25.2	P MIS
Leuchte et al. [107]	*Z Orthop*	Retrospective	7.0	16		59.7	26.7	AL MIS
				16		62.6	28.6	L
Lawlor et al. [108]	*Clin Rehab*	Randomised	1.5	109	55.0	67.4	28.2	P MIS
				110	47.3	65.9	28.9	P
Malek et al. [109]	*Bone Joint Surg*	Retrospective	18.1	265	55.8	70.8	28.5	A
				183	53.0	70.0	29.0	P
Martin et al. [110]	*Arthroplasty*	Randomised	12.0	42	71.4	66.7	30.6	AL MIS
				41	65.9	63.1	29.4	L
Martin et al. [111]	*Arthroplasty*	Retrospective	6.0	47	65.0	63.0	28.5	A
				41	55.0	57.0	34.1	P
Mazoochian et al. [112]	*Arch Orthop Trauma Surg*	Randomised Prospective	3.0	26	56.0		26.6	LA MIS
				26	65.4		26.4	LA/ Bauer
Migliorini et al. [113]	*Surgeon*	Restrospective	24	70	78.6	67.2	26.9	AL MIS
				70	84.3	66.1	27.6	AL
Mjaaland et al. [114]	*Clin. Ortho Rel Reas*	Randomised	24.0	84	70.0	67.0	28.0	A
				80	62.0	66.0	28.0	L
Müller et al. [115]	*Arch Orthop Trauma Surg*	Randomised	12.0	24	50.0	66.0	28.0	AL MIS
				20	60.0	64.0	26.0	L
Nakata et al. [116]	*Arthroplasty*	Retrospective	12.0	99	83.8	62.9	22.9	A
				96	86.5	65.6	23.3	P MIS
Ogonda et al. [9]	*Bone Joint Surg*	Randomised	1.5	109	55.0	67.4	28.2	P MIS
				110	47.3	65.9	28.9	P
Palan et al. [117]	*Clin Orthop Rela Res*	Prospective	60.0	699	60.9	68.4	27.5	AL
				390	64.1	67.4	27.0	P
Petis et al. [118]	*Arthroplasty*	Prospective	0.1	40	62.5	66.9	27.9	A
				40	65.0	66.7	28.2	P
				40	65.0	65.5	29.1	L
Poehling-Monaghan et al. [63]	*Clin Orth Rel Res*	Prospective	2.0	50	48.0	63.0	31.0	A
				50	56.0	63.0	30.0	P MIS
Pogliacomi et al. [119]	*Hip Int*	Retrospective	12.0	30	53.3	68.6	27.3	L
				30	50.0	67.7	27.0	A MIS
Pospischill et al. [3]	*Bone Joint Surg*	Randomised	3.0	20	60.0	61.9	25.7	AL MIS
				20	40.0	60.6	25.7	L
Queen et al. [120]	*Arthroplasty*	Prospective	12.0	10	n.a	60.0	26.6	Direct lateral
				10	n.a	57.0	26.3	P
				10	n.a	57.6	28.8	AL
Radoicic et al. [121]	*Int Orthop*	Prospective	6.0	21	61.9	60.9		A
				21	61.9	60.9		P
Rathod et al. [122]	*Arthroplasty*	Retrospective	12.0	11	45.5	58.0	25.9	DA
				11	45.5	61.8	25.4	P
Reichert et al. [123]	*BMC Musculoskelet Disorders*	Randomised	12.0	73	43.8	62.5	28.3	A
				50	52.0	62.2	28.7	L
Rittmeister et al. [124]	*Orthopäde*	Retrospective	0.2	76	69.7	60.0	28.0	P MIS
				76	69.7	65.0	27.0	AL
Rodriguez et al. [125]	*Clin Orth Related Res*	Prospective	12.0	60	53.3	60.0	27.0	A
				60	56.7	59.0	28.0	P
Rosenlund et al. [126]	*Acta Orthop*	Randomised	12.0	38	31.6	60.0	27.0	L
				39	33.3	62.0	28.0	P
Rykov et al. [61]	*Arthroplasty*	Randomised	1.5	23	65.2	62.8	29.0	A
				23	52.2	60.2	29.3	PL
Schleicher et al. [127]	*Acta Orthop*	Prospective	6.0	64	68.7	69.1	28.8	L
				64	75.0	68.3	27.1	P MIS
Sendtner et al. [128]	*Arch Orthop Trauma Surg*	Prospective	12.0	74	32.4	68.1	28.8	A MIS
				60	30.0	67.9	29.1	L (Bauer)
Sershon et al. [129]	*Arthroplasty*	Randomised	98.0	31	67.7	73.4	28.2	P MIS
				32	71.9	70.9	28.7	2 incision
Shitama et al. [10]	*Int Orthop*	Randomised	6.0	15	85.3	61.7	23.2	MIS TL
				19	85.3	58.3	23.2	MIS PL
				8	85.7	53.4	23.0	Translateral
				20	85.7	61.3	23.0	PL
Spaans et al. [130]	*Acta Orthopaedica*	Prospective	12.0	46	47.8	69.0	25.0	DAA MIS
				46	69.6	68.0	29.0	PL
Sugano et al. [131]	*Orthop Clin N Am*	Prospective	24.0	39	92.3	57.0	23.0	P MIS
				33	87.8	56.0	23.0	A MIS
Szendri et al. [132]	*Int Orthop*	Randomised	3.0	38		64.0	26.0	L MIS < 10 cm
				43		62.0	28.0	MIS > 10 cm
				21		57.0	29.5	> 14 cm L
Takada et al. [133]	*J Orthop Sci*	Randomised	12.0	30	86.7	62.6	24.4	DL
				30	86.7	62.6	24.4	AL
Taunton et al. [134]	*Arthroplasty*	Randomised	12.0	27	51.9	66.4	29.2	P MIS
				27	55.6	62.1	27.7	A
Varelaegochaega et al. [8]	*Eur J Orth Sur Traumatol*	Randomised	60.0	25	52.0	64.8	28.3	MIS L
				25	52.0	63.8	27.8	L
Vicente et al. [135]	*Clinics*	Retrospective	6.0	34	32.2	50.0	27.0	P MIS
				42	38.1	57.0	27.0	L
Wayne et al. [136]	*Orthop Rev*	Prospective		100	66.0	68.0	27.0	L
				100	71.0	68.0	26.6	A MIS
Wohlrab et al. [137]	*Z Orthop*	Retrospective	3.0	27	59.3	58.8	27.2	P MIS
				23	52.2	61.9	29.3	L
Wright et al. [138]	*Arthroplasty*	Randomised	60.0	42		64.2	24.4	MIS L
				42		65.0	28.3	L
Yang et al. [139]	*Ir J Med Scien*	Randomised	36.0	55	52.7	59.5	23.1	AL MIS
				55	45.5	55.8	22.4	PL
Zawadsky et al. [140]	*Arthroplasty*	Retrospective	0.5	50	70.0	56.0	27.9	P MIS
				50	56.0	60.8	28.6	A
Zhao et al. [141]	*Arthroplasty*	Randomised	6.0	60	60.0	64.9	24.4	A
				60	56.0	62.2	25.6	PL

### Outcomes of interest

Female gender was strongly associated with lower cup anteversion (*r* =  − 0.52; *P* = 0.0002). Older age was moderately associated with reduced surgical time (*r* =  − 0.28; *P* = 0.01), and with greater VAS (*r* = 0.42; *P* = 0.02) and WOMAC scores (*r* = 0.52; *P* = 0.009) at last follow-up. Greater BMI at baseline was associated with greater cup anteversion (*r* = 0.47; *P* = 0.0009), greater OHS at last follow-up (*r* = 0.47; *P* = 0.04), longer surgical duration (*r* = 0.20; *P* = 0.04), greater leg length discrepancy (*r* = 0.47; *P* = 0.02), and greater rate of deep infection (*r* = 0.44; *P* = 0.04). Greater VAS at baseline was associated with greater VAS at last follow-up (*r* = 0.98; *P* < 0.0001), greater overall estimated blood lost (*r* = 0.11; *P* = 0.01), and lower value of HHS (*r* =  − 0.98; *P* = 0.0005). Greater OHS at baseline was associated with post-operative greater VAS (*r* = 0.88; *P* = 0.01). Greater WOMAC at baseline was associated with lower cup anteversion (*r* = 0.89; *P* = 0.009) and greater VAS at last follow-up (*r* = 0.88; *P* = 0.02). Greater HHS at baseline was associated with shorter hospitalisation (*r* = 0.50; *P* = 0.001). No other statically significant associations were evidenced. The results of the multivariate analyses are shown in greater detail in Table 2.

**Table 2:** Overall results of the multivariate analyses

	Sex—baseline		Age—baseline		BMI—baseline		VAS—baseline		OHS—baseline		WOMAC—baseline		HHS—baseline	
	*r*	*P*	*r*	*P*	*r*	*P*	*r*	*P*	*r*	*P*	*r*	*P*	*r*	*P*
Cup inclination	− 0.16	0.2	− 0.05	0.7	− 0.15	0.2	0.45	0.5	− 0.32	0.3	− 0.23	0.5	− 0.02	0.9
Cup anteversion	− 0.53	0.0002	0.20	0.2	0.47	0.001	1.00	1.0	0.04	0.9	− 0.89	0.009	0.07	0.8
Stem alignment	− 0.04	0.9	− 0.15	0.5	− 0.07	0.7			0.29	0.4	− 0.81	0.1	− 0.39	0.2
VAS	− 0.26	0.2	0.42	0.02	0.17	0.4	0.98	0.00001	0.88	0.01	0.88	0.02	0.16	0.5
OHS	− 0.06	0.8	0.52	0.009	0.47	0.04			− 0.20	0.5	0.91	0.1	− 0.57	0.1
WOMAC	0.04	0.9	− 0.47	0.1	− 0.45	0.1			0.65	0.1	0.75	0.1	0.81	0.3
HHS	− 0.08	0.5	− 0.28	0.01	− 0.01	0.9	− 0.98	0.0005	− 0.34	0.3	− 0.66	0.1	0.36	0.05
Surgical time	0.07	0.5	− 0.19	0.1	0.20	0.04	0.65	0.1	− 0.03	0.9	0.53	0.1	0.07	0.6
Estimated blood lost	0.15	0.3	− 0.06	0.7	− 0.15	0.3	0.11	0.01	0.18	0.6	0.50	0.1	0.19	0.3
Leg length difference	0.11	0.6	− 0.20	0.3	0.47	0.02	− 1.00	1.0	0.33	0.4	− 0.08	0.9	− 0.43	0.1
Hospitalisation	0.20	0.1	0.11	0.3	− 0.22	0.1	0.94	0.1	− 0.51	0.2	− 0.55	0.2	− 0.50	0.001
Dislocation	− 0.17	0.3	0.13	0.4	− 0.05	0.8	1.00	1.0	0.44	0.3			− 0.29	0.2
Revision	0.10	0.6	0.36	0.1	0.05	0.8	0.90	0.3	− 0.37	0.4	− 1.00	1.0	0.13	0.7
Deep infection	− 0.08	0.7	0.30	0.2	0.44	0.04	0.61	0.6	− 0.98	0.2			0.00	1.0
Aseptic loosening	0.07	0.9	− 0.28	0.5	− 0.51	0.1			0.42	0.6	− 1.00	1.0	0.24	0.7
Fractures	− 0.06	0.7	0.08	0.6	− 0.04	0.8	0.41	0.5	− 0.24	0.5	0.54	0.3	0.24	0.3

## Discussion

According to the main findings of this systematic review, older age and greater BMI were negative prognostic factors for the outcome of MIS THA. The analyses of the PROMs suggested that the clinical outcome is strongly related to the preoperative status of the patient.

The role of age is controversial. Previous studies observed greater improvements in pain and function after THA in older patients [28–32], while others demonstrated no substantially better clinical outcome [33–35]. Muscle trauma in older patients via MIS approach should be minimised to improve the functional outcome [36]; [37]. The greater rates of complication and overall worse outcome in patients with BMI exceeding 30 kg/m^2^ has been extensively investigated. The negative influence of obesity for THA was likewise demonstrated by previous studies [38–42]. Lower PROMs scores, longer hospitalisation, greater blood loss, higher rate of wound complications, deep venous thrombosis, and infection are the most common complications [38–42]. The reduced access to the operative field, extensive bleeding surfaces, and greater force of retraction do not seem to have relevant influence in terms of component malpositioning, prolonged operative times, and higher intraoperative blood loss in obese patients during MIS THA [43–45]. Timing of mobilisation, length of hospitalisation, and functional outcome were similar between obese and non-obese patients [45], and obese patients should be strongly encouraged to lose weight prior to THA. However, it has been hypothesised that only bariatric surgery in obese patients before arthroplasty could realistically cut down complications [46–51]. Female gender was strongly associated with lower cup anteversion. However, the native anteversion of the femoral neck differs between males and females, with a physiological mean acetabular anteversion of approximately 16° and 12.5°, respectively [52]; [53]. Gender-specific anatomical differences increase data variability and may lead to inconsistency in results. Furthermore, to investigate the cup anteversion malpositioning, the acetabular inclination angle must also be considered [54]. MIS THA has been advocated to reduce consumption of pain medications [15]; [55]. High post-operative pain negatively influences the clinical outcome and predisposes to chronic pain [56]. Greater post-operative pain and the fear of it may lead to immobility and delayed post-operative rehabilitation [57]; [58]. The reduced surgical incision and tissues trauma may reduce pain and the blood loss and represent the main motivation to opt for a MIS approach [2]; [12]; [59]. However, previous studies did not evidence clinically relevant difference between standard and MIS THA in pain and total estimated blood lost [2]; [12]; [59]. The reduced damage to the tissues of the MIS approaches has been advocated to improve functional outcomes, and inflammation markers have been employed to evaluate soft tissue damage [60]; [61]. Recent evidence showed no significant differences in serum markers of muscle damage and inflammation between minimally and standard THA approaches [62]. Furthermore, serum markers did not predict early pain/function after THA and were not associated with early functional outcomes either in-hospital or post-discharge [63].

The present systematic review certainly has limitations. The current published literature lacks high-quality studies which analysed the influence of prognostic factors for MIS THA, and the limited number of included studies represent an important limitation. Several studies (277 of 683, 41%) were excluded for redundancy. To improve data pooling, both prospective and retrospective studies were included in the analysis, which inevitably increases the risk of selection bias. A limitation of this study is represented by the relative short length of the mean follow-up. Half of studies were randomised, but, given the overall lack of blinding methods, the risk of detection bias was moderate-high. Furthermore, the different approaches for THA were not considered separately, nor were the different implant designs [64–81]. Given these limitations, data from the present study must be interpreted with caution. Strengths of this work were the study size, the description of diagnosis and surgical techniques which were stated and adequate. Another strength of the present systematic review is the comprehensive nature of the literature search and rigorous assessment of methodological quality of the current available data.

## Conclusion

Older age and greater BMI were negative prognostic factors for MIS THA. The analyses of the PROMs suggested that the clinical outcome is strongly related to the preoperative performance status of the operated patients. There is no compelling evidence that MIS THA offers advantages over traditional approaches, especially when modern analgesia techniques and accelerated rehabilitation programmes are considered.

## Data Availability

The datasets generated during and/or analysed during the current study are available from the corresponding author on reasonable request.

## Abbreviations

MIS: Minimally invasive surgery
THA: Total hip arthroplasty
PROMs: Patient-reported outcome measures
PRISMA: Preferred reporting items for systematic reviews and meta-analyses
VAS: Visual Analogue Scale
OHS: Oxford Hip score
WOMAC: Western Ontario and McMaster Universities Osteoarthritis Index
HHS: Harris Hip Score
SD: Standard deviation
IQR: Interquartile range
